# Observational evidence of wildfire-promoting soil moisture anomalies

**DOI:** 10.1038/s41598-020-67530-4

**Published:** 2020-07-03

**Authors:** Sungmin O, Xinyuan Hou, Rene Orth

**Affiliations:** Department of Biogeochemical Integration, Max Planck Institute for Biogeochemistry, 07745 Jena, Germany

**Keywords:** Hydrology, Natural hazards

## Abstract

Wildfires can destroy property and vegetation, thereby threatening people’s livelihoods and food security. Soil moisture and biomass are important determinants of wildfire hazard. Corresponding novel satellite-based observations therefore present an opportunity to better understand these disasters globally and across different climate regions. We sampled 9,840 large wildfire events from around the globe, between 2001 and 2018, along with respective surface soil moisture and biomass data. Using composites across fire events in similar climate regions, we show contrasting soil moisture anomalies in space and time preceding large wildfires. In arid regions, wetter-than-average soils facilitate sufficient biomass growth required to fuel large fires. In contrast, in humid regions, fires are typically preceded by dry soil moisture anomalies, which create suitable ignition conditions and flammability in an otherwise too wet environment. In both regions, soil moisture anomalies continuously decrease in the months prior to fire occurrence, often from above-normal to below-normal. These signals are most pronounced in sparsely populated areas with low human influence, and for larger fires. Resolving natural soil moisture–fire interactions supports fire modelling and facilitates improved fire predictions and early warning.

## Introduction

Wildfires are defined as uncontrolled fires that combust vegetation material^1^. While they are prevalent in ecosystems and societies around the globe, they have substantial negative impacts. Wildfires can be disastrous for local ecosystems and communities as they destroy vegetation and property, threatening people’s livelihoods and food security^2,3^. They are also a significant source of CO_2_ and air pollutants^4,5^. However, some positive impacts can also occur. Wildfires yield nutrients and space to facilitate ecosystem development and adaptation^6^. In this way, they contribute to global vegetation productivity and biodiversity, and consequently, improving ecosystem services; e.g., mitigating weather extremes like heat waves or droughts or removing CO_2_ from the atmosphere^7–11^.

Given the significance of wildfires, many studies have explored their controlling factors. They report complex relationships between wildfires and variables such as climate, vegetation type, and human influence, where their respective importance varies between regions or seasons^12–14^. While fires can be ignited by humans or lightning^1,15^, climate influences the possible fire spread and size^16,17^, and hence the potential implications of wildfires^18^. In this context, soil moisture has been identified as a key variable for understanding and predicting wildfire hazard^1,19–22^. Soil moisture, defined as the water contained in the unsaturated soil zone^23^, not only influences vegetation growth conditions and consequently the accumulation of wildfire fuel, but also determines the vegetation moisture content and hence the flammability of the vegetation^1,20,24^. However, owing to a lack of global observation-based soil moisture data, previous studies have focused on case studies at local to regional scales^1, 20–22^, employed model-based soil moisture information, or used drought indices such as Keetch–Byram drought index or Standardized Precipitation Index^22,25,26^. Benefitting from recent advances in the satellite-based derivation of surface soil moisture, we perform a global analysis of the role of soil moisture in the occurrence of large wildfires. This allows us to explore potential differences in pre-fire soil moisture conditions across cold and warm, as well as humid and arid regions.

In particular, we consider the ESA CCI satellite soil moisture product^27–29^ and focus on the surface soil moisture anomalies preceding the largest local wildfires during the study period 2001–2018. This involves the identification of fire-promoting soil moisture anomaly patterns, i.e. signs, magnitudes and lead times of soil moisture anomalies typically preceding the large fires. Fire activity throughout this study is characterised using the burned area product generated from the Moderate Resolution Imaging Spectroradiometer (MODIS)^30^. In each 0.25**°** × 0.25**°** grid cell, we only consider the largest burned area on record, as we assume this is associated with most informative soil moisture patterns. In order to identify a physical link between the soil moisture patterns and fire occurrences, we additionally employ vegetation optical depth (VOD) as a proxy for vegetation biomass^31^. Finally, enabled by the large amount of employed global data, we focus on low human population areas only by selecting grid cells with population densities below the 25th percentile of all available global grid cells, which corresponds to 1.68 people/km^2^. This approach allows the exclusion of areas where human-made infrastructure covers significant space, and where human fire suppression obscures natural soil moisture–fire interactions.

## Results

### Global distribution of largest wildfires

**Figure 1:**
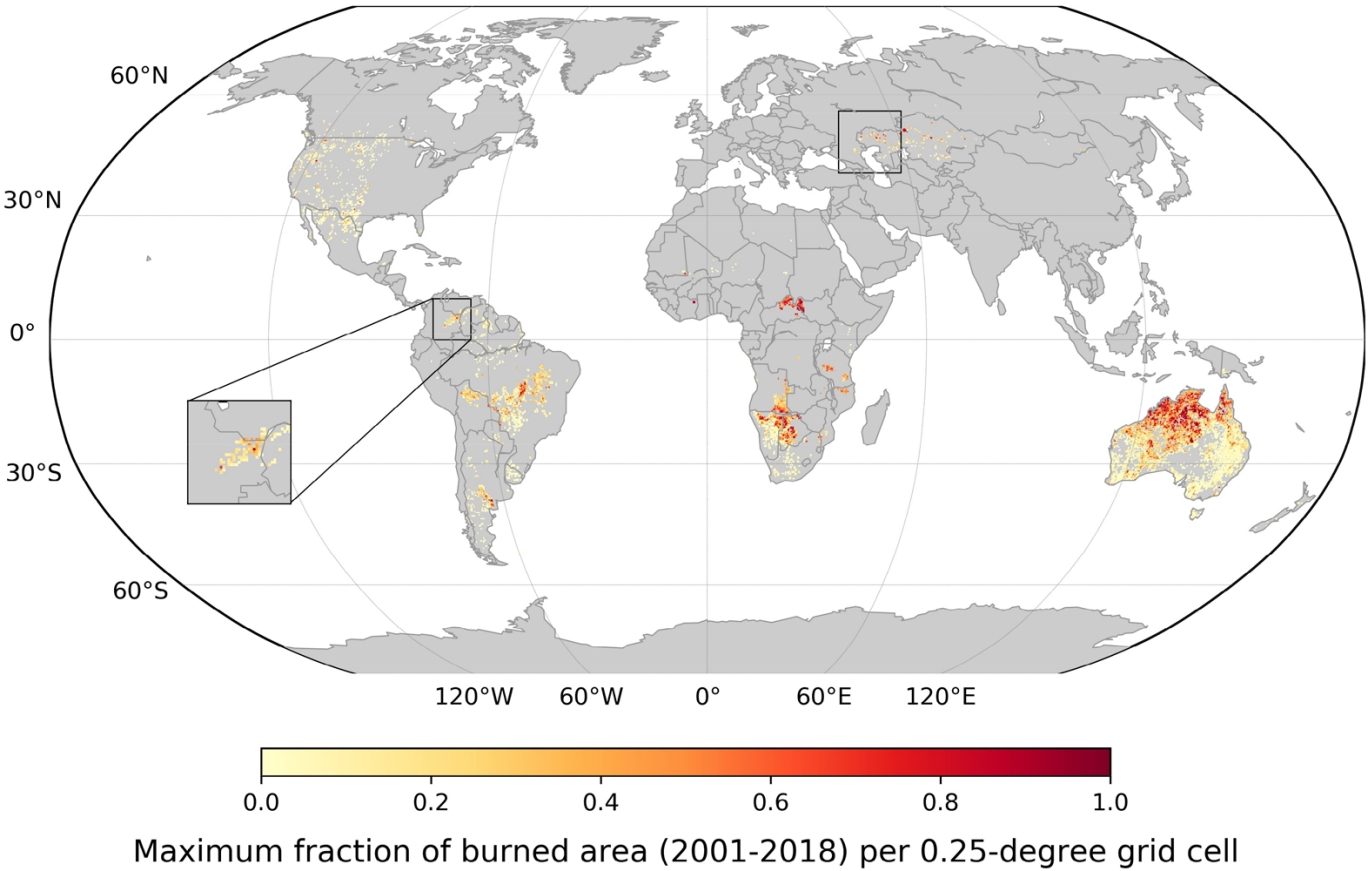
Largest wildfires located in (sub-)tropical regions. Maximum monthly burned area during 2001–2018, as a fraction to the total land area per 0.25 degree grid cell. Grid cells selected considering population density, data quality, and availability of multiple data streams used in this study (see Methods). Black squares show example countries showing spatial variations in the burned area size along the borders. The map is created using the Matplotlib basemap v1.3.0 toolkit^55^.

After filtering out the grid cells that have any missing soil moisture data during the 5 months preceding the largest fires, and selecting the low population density areas, 9,840 grid cells are left for our global analysis. The corresponding largest burned areas as a fraction of burned area to the total land area per 0.25° grid cell are shown in Fig. 1. Overall largest fires are diagnosed in tropical and subtropical regions, with a pronounced respective north–south gradient across Australia. Discarding cold regions where the soil moisture data is of potentially lower quality limits the use of data over northern regions including e.g., boreal forests^27^. Nonetheless, suitable grid cells for the subsequent analyses are distributed across continents and latitudes, spanning multiple climate regimes. Largest burned areas are found mostly in the tropics, likely induced by high biomass and strong seasonal variations in precipitation resulting in dry (or drier) seasons. As expected, many grid cells retained after the filtering are from flammable forested areas in western North America. The lower fire occurrence in the Mediterranean compared to similar climate regions such as western North America is due to the exclusion of highly populated areas, which implies different regional land uses^32^. Often, some variations in data availability and fire size are found along country borders such as for Kazakhstan and Colombia (see Fig. 1), probably owing to different population densities and/or land management practices^33^.

### Fire-promoting soil moisture patterns

**Figure 2:**
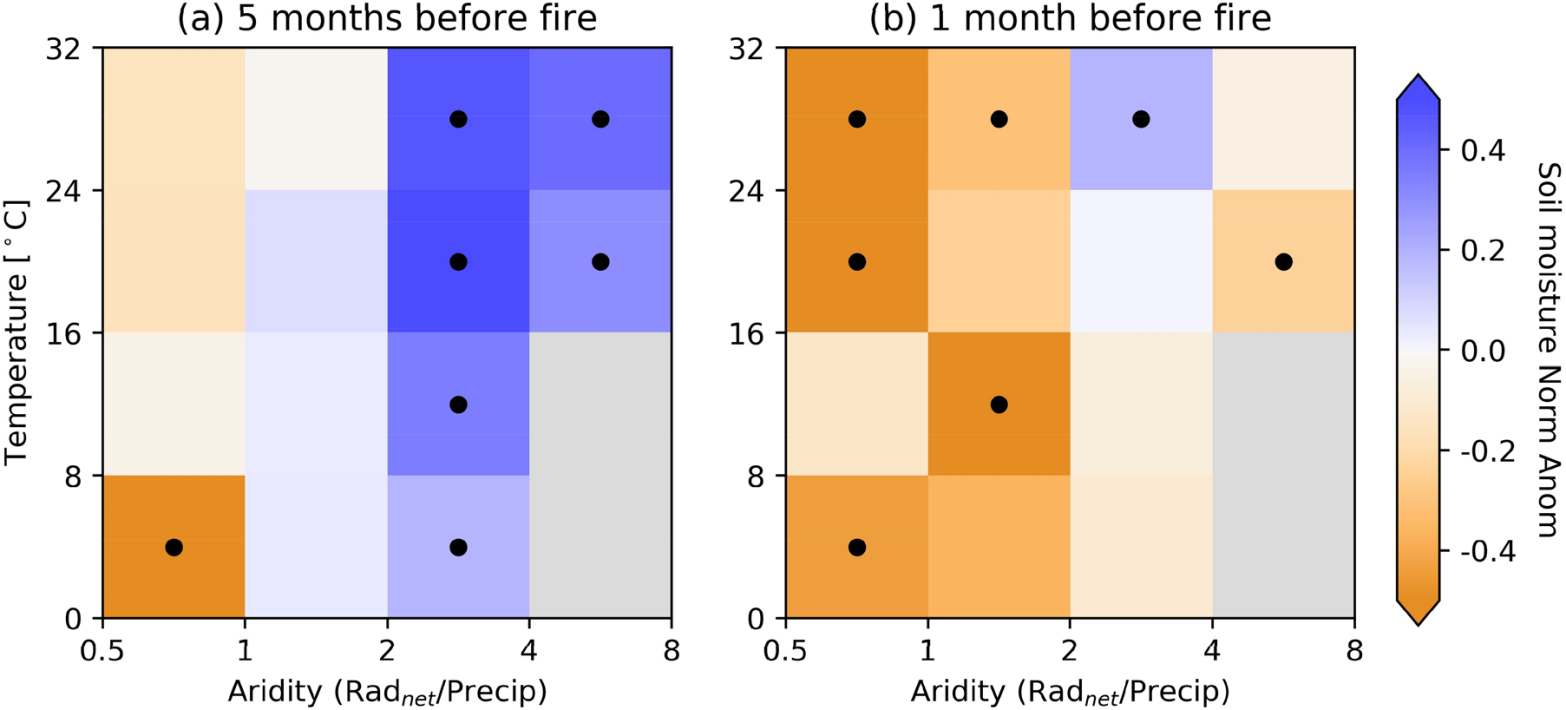
Consecutive wet and dry soil moisture conditions promote wildfires. Normalised soil moisture anomalies at (**a**) 5 months and (**b**) 1 month before the month with the largest burned area. Grid cells are grouped with respect to long-term temperature and aridity. Median values across grid cells in each box are shown. Boxes with less than 25 grid cells are discarded and shown in gray. Black dots within the boxes denote significant anomalies at the 90%-level.

We group all grid cells with respect to their long-term mean temperature and aridity. The aridity is computed as net radiation (converted to equivalent evaporation) divided by precipitation^34^. Then we assess mean normalised soil moisture anomalies preceding the largest wildfires for respective grid cells within a similar climate. Here we focus on times long (5 months) and shortly (1 month) before the fires to evaluate the potential of soil moisture data as a predictor of wildfires. We find significant wet anomalies 5 months before the fires in dry regions, and significant dry anomalies 1 month before the fire in wet regions (Fig. 2). Although the data is not evenly distributed, Fig. S1 shows that there are still a considerable amount of grid cells across most climate regimes, allowing us to achieve robust results. Anomalies are defined as a deviation from the long-term monthly average (2001–2018) and then normalised by the respective month-of-year standard deviation (z-score). We also assess the significance of the observed anomalies by comparing with anomalies recomputed from randomly selected non-fire months (see Methods).

Overall, soils are wet(ter) longer ahead of the fire, and getting drier towards the fire. As a result, in many climate regimes, consecutive above-normal (wet) and below-normal (dry) soil moisture anomalies are observed prior to the largest wildfires. In Fig. S2, we repeat this analysis with VOD observations^31^, which is a measure of the attenuation of microwaves as they pass through the vegetation canopy, and consequently related to above-ground biomass. It shows that the wet soils favour biomass (i.e., potential fire fuel) accumulation, while the subsequent drying limits further biomass increase and likely also vegetation water content^35–37^, leading to higher flammability.

Further, while the results in Fig. 2 are similar across temperature classes, there is a contrast between the humid (aridity ≤ 2) and arid (aridity > 2) regions. In arid regions the magnitude of the wet soil moisture anomalies 5 months before the fires is greater than in humid regions, compensating for the water-limiting vegetation growth conditions. The observed normalised anomalies range from 0.20 to 0.55 in arid regions, whereas − 0.81 to 0.07 (mostly non-significant) in humid regions. In humid regions, however, the magnitude of the dry soil moisture anomalies shortly before the fire is greater (− 0.13 to − 0.68), contributing to the dry-out of the vegetation. Correspondingly, most positive biomass anomalies are found in arid regions and longer before the fire, while most negative anomalies are found in humid regions directly before fire occurrence (Fig. S2); this is also consistent with an earlier study^38^ investigating the global fire and productivity relationship. While our study focuses on different climate regimes, such contrasting soil moisture behaviors before fires were also observed between different seasons by Krueger et al^39^. They showed with data from Oklahoma that the probability of large wildfires was increased by negative soil moisture anomalies at the time of the fire during the growing season, whereas dormant-season wildfire probability was increased by positive soil moisture anomalies in previous months. Most of the fires we investigate (~ 83%) occurred during the growing season.

### Temporal evolution of climate and biomass anomalies before and after fires

**Figure 3:**
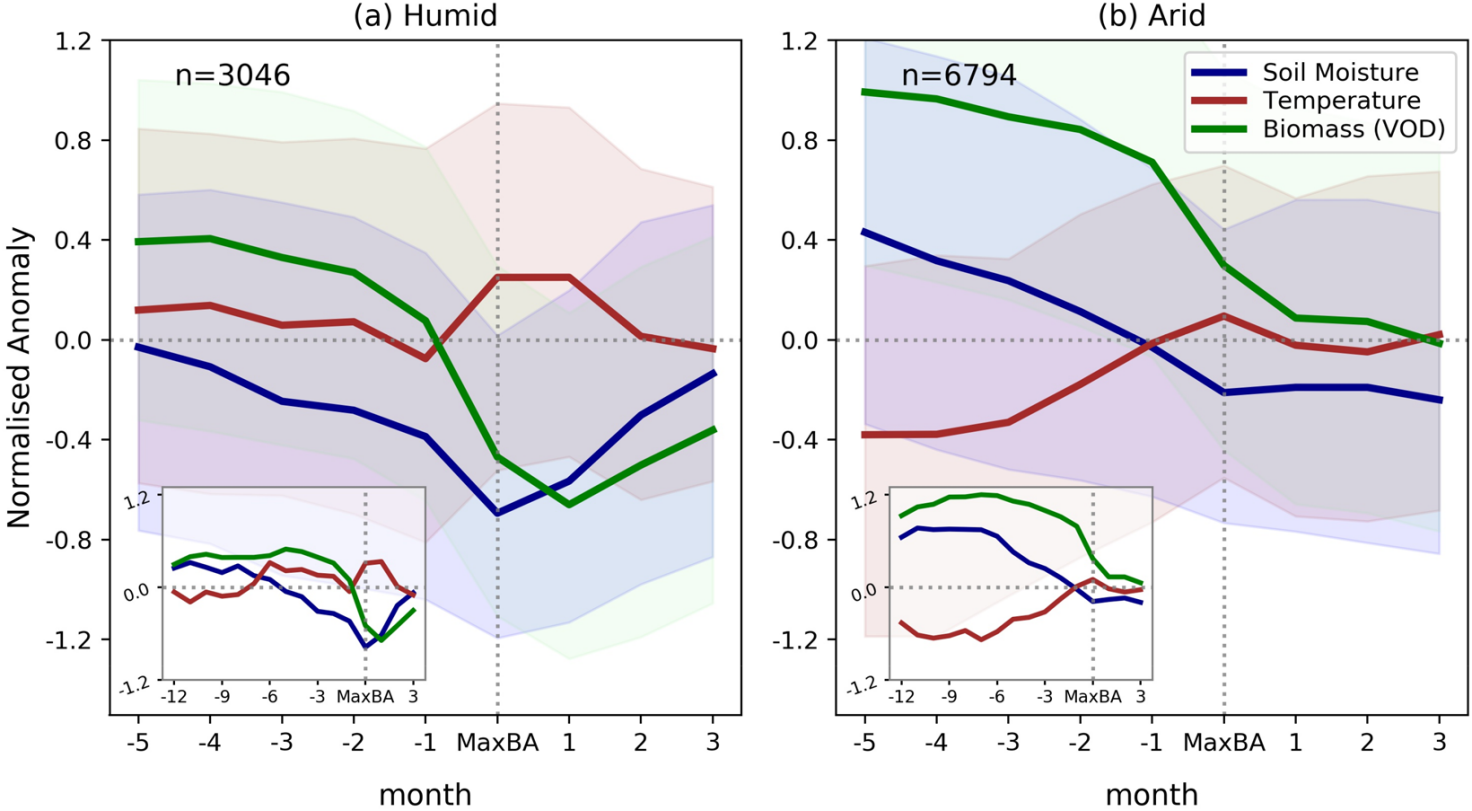
Jointly decreasing soil moisture and biomass before fire outbreaks. Temporal evolution of normalised anomalies of soil moisture, temperature, and biomass (VOD) before and after wildfire outbreaks in (**a**) Humid (aridity ≤ 2) and (**b**) Arid regions (aridity > 2). Lines show median values and the shading denotes the interquartile range across the considered grid cell values. The number of grid cells in both regions is denoted by ‘n’. Insets show the temporal evolution extending to 12 months before the fire occurrence.

The distinct role of soil moisture in humid versus arid regions is also apparent in the overall temporal evolution of climate and biomass anomalies around the times of the fires (Fig. 3). Soil moisture anomalies are continuously decreasing before the fire outbreaks, but at a generally wetter level in arid regions compared with the humid regions. Fires usually do not last longer than one month (Fig. S3), and afterwards soil moisture and biomass anomalies are relatively quickly recovering in humid regions, but not in arid regions. This might be due to more frequent rainfall in humid regions, which can more rapidly compensate existing soil moisture deficits^40^. The similarity of the soil moisture and biomass anomaly evolutions around fire events indicates that soil moisture is a major driver of fire-promoting biomass anomalies. In addition, the fires influence biomass evolution and cause a clear decrease. This highlights that fires can be equally important as soil moisture for large-scale biomass dynamics. By contrast, the corresponding temperature anomalies are mostly weaker and not corresponding to the same extent with the biomass anomalies. These results are robust across several state-of-the-art products estimating vegetation biomass from VOD observations (Fig. S4). Further extending the temporal range before the fire occurrence, we find that fire-related soil moisture and biomass anomalies are present even one year before the burned area peaks (insets in Fig. 3; see also refs.^17,39,41^). The soil moisture anomalies in both humid and arid regions begin to decrease around 5 to 6 months before the fires, continuously until the fire outbreaks. As also shown in Fig. 3, the biomass evolution overall follows the soil moisture evolution, even though some time lag is observed in humid regions.

While these results are based on averaged composite temporal evolutions across many grid cells, it should be noted that there is substantial variability across the individual grid cells. This is shown by the interquartile range displayed in Fig. 3. It indicates that large fires can also occur with climate and biomass anomalies which are rather different from the described mean fire-related climate and biomass anomalies. This reflects that in addition to soil moisture and biomass anomalies, wildfires are controlled by many other factors, making them complex phenomena^14,42^. Additional controlling factors include soil and vegetation types and the corresponding composition across the ecosystem^1,38,43^; fire characteristics might further vary with respect to burned area size and the role of humans^44^. The latter factors are further investigated in the following section.

### Inter-related controls of large fire occurrence

**Figure 4:**
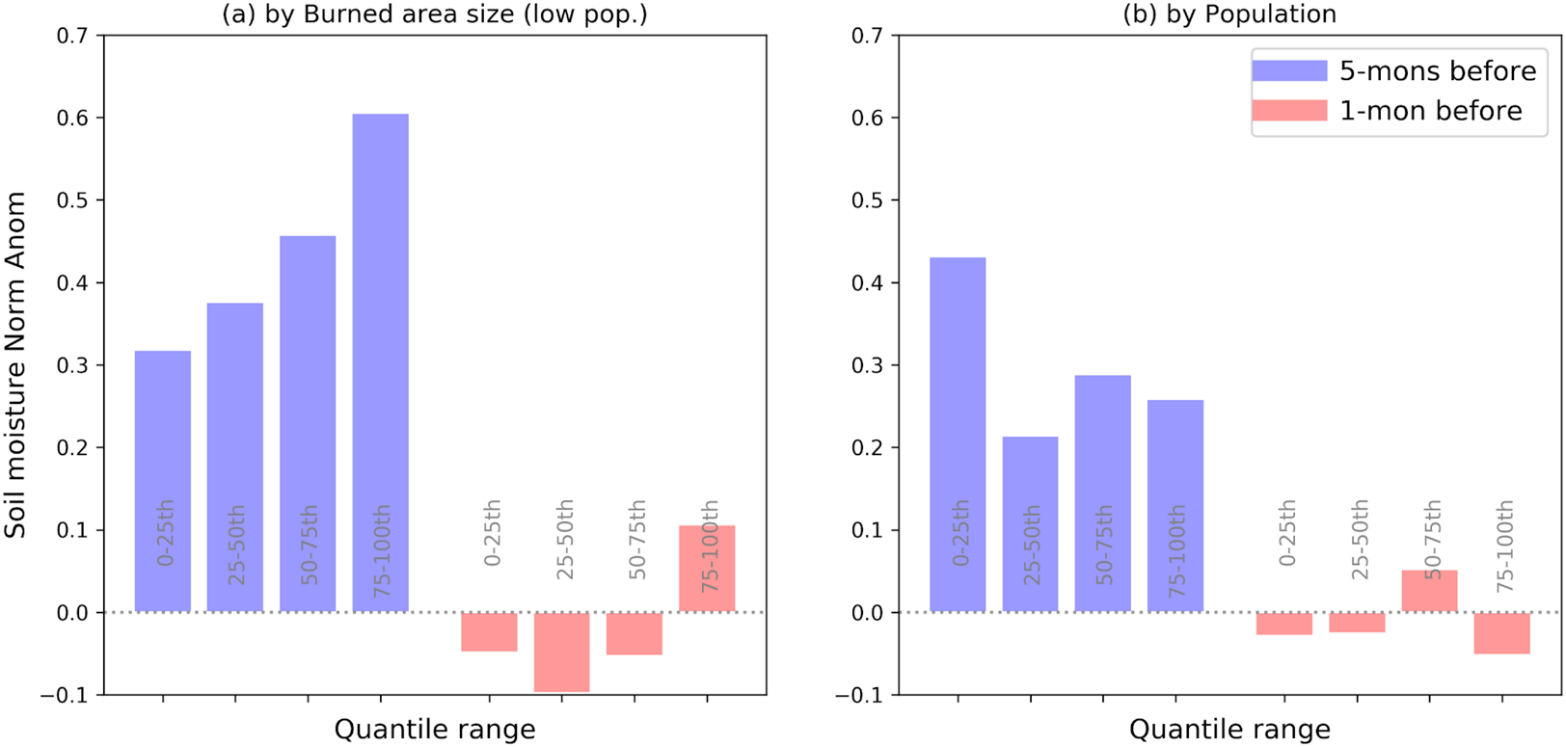
Stronger role of soil moisture at low population and for large fires. Soil moisture conditions at 5 months and 1 month before the maximum burned area are computed for different (**a**) burned area sizes, and (**b**) population densities. Only arid regions (aridity > 2) are considered.

We repeat the previous analysis of fire-preceding soil moisture anomalies for subsets of different maximum burned area sizes across the considered grid cells shown in Fig. 1. The results for arid regions are shown in Fig. 4a. The wet soil moisture anomalies 5 months before the fires are generally more pronounced for larger fires, suggesting that the characteristic soil moisture anomalies long ahead of the fires are relevant to fire size. This pattern is still present but weaker at 1 month before the fire. This indicates that fire ignition conditions, as indicated by soil moisture 1 month before the fire, are more similar across fires of different size, in contrast to pre-fire biomass accumulation, as indicated by soil moisture 5 months before the fire. Moving beyond fire size, we also test the role of population density in the soil moisture-fire interactions. For this purpose, we re-conduct the previous analysis with all 39,363 grid cells where soil moisture data is available and group the grid cells into quartiles. Fire-related soil moisture anomalies 5 months before the fires are higher in sparsely populated areas (0–25th percentile) than in the more populated areas (Fig. 4b). However, no systematic variation of the soil moisture anomalies 5 months before the fires is observed across the other population density classes. This indicates that (near-)natural dynamics between the hydro-climatic conditions and fire behaviour can only be observed in the most sparsely populated areas. In more populated areas, human-made infrastructures require space and replace vegetation. Further, in these areas, fire suppression is likely more prevalent^45,46^.

While these analyses focused on arid regions, we find less pronounced soil moisture patterns with respect to burned area size and population in humid regions (Fig. S5). This indicates a secondary role of soil moisture in these regions. This is probably due to the fact that moisture is (mostly) not limiting and therefore only weakly controlling biomass dynamics in a wet climate, while it is still relevant to create suitable ignition conditions.

## Discussion

Overall, our observation-based analyses have revealed characteristic soil moisture anomalies prior to large wildfires which can inform the anticipation of these events. At the considered large spatial scales, soil moisture has a more significant influence on fire-promoting vegetation conditions than temperature^39^. Soil moisture indirectly affects subsequent fire probability and size in two ways: (1) it controls biomass growth and hence fire fuel availability, and (2) it determines vegetation moisture content and hence respective flammability. The global focus of our analysis provides unique insights – the first pathway is more important in arid regions, while the second pathway is more relevant in humid regions. It should be noted that such contrasting soil moisture patterns can also be observed in time as opposed to space, for example with seasonal variations (e.g., growing vs dormant seasons^39^).

Given the multitude of potential fire controls and their complex interplay, it is difficult to isolate the role of soil moisture. While this limitation does not affect the overall validity of our findings, it needs to be taken into account when interpreting the results. For instance, the high uncertainty across grid cells shown in Fig. 3 is likely due to the complex nature of fire occurrence which is driven by multiple factors with varying spatio-temporal properties. We take this into account by filtering the data to focus on sparsely populated areas in the first place and by comparing the results across different climate regimes. Yet, this complexity limits more detailed analyses at local level. In addition, Fig. 4 indicates the potential role of confounding factors such as the interlinked soil moisture and population density on wildfire hazard. We counter this by including biomass anomalies in the analyses and repeating them for both humid and arid regions, to demonstrate the physical link between soil moisture, vegetation biomass, and fire occurrence. Finally, it should be noted that the identified patterns of soil moisture and biomass anomalies corresponding to large wildfires do not directly cause the fires: rather, they determine the possibility of ignition and the size of burned areas.

Our main findings based on satellite observations can inform the fire modelling community and enable respective model enhancements^47^. In turn, this will contribute to improved fire forecasting, especially since fire-promoting soil moisture and biomass anomalies develop very slowly and therefore have a long lead-time. The global perspective on soil moisture–vegetation–fire interactions provided here confirms and integrates previous studies performed at smaller spatial scales^17,48–50^, thereby facilitating more reliable local-scale fire predictions and early warning across the globe.

## Methods

### Fire data sampling

We use monthly burned area data from the MODIS Fire_cci v5.1 dataset provided by the European Space Agency Climate Change Initiative^30^ (ESA CCI; https://geogra.uah.es/fire_cci/). The data has a spatial resolution of 0.25° × 0.25°. We only use burned area data from grid cells with a fraction of observed area higher than 80%. In each of these grid cells, we select the highest monthly burned area value during the study period 2001–2018 for the main analyses. To enable the derivation of robust temporal soil moisture evolutions, we generally discard grid cells where any soil moisture value between 5 months before and 1 month after the maximum burned area is missing. We also discard grid cells with long-term average (2001 to 2018) temperature lower than 0 °C to exclude lower-quality soil moisture data impacted by freezing processes, e.g., at high latitudes and in boreal forest environments^27^^.^

### Data processing

All data used in this study are averaged to monthly means and re-gridded to 0.25° × 0.25° resolution in order to match the spatio-temporal resolution of the burned area data. In the case of soil moisture and VOD, monthly mean values are only computed if at least 15 days of data are available in a monthly period. We use the 18-year monthly averages and standard deviations to compute the normalised anomalies of soil moisture, VOD, and temperature.

### Assessing statistical significance

Significance of the observed anomalies in Fig. 2 is assessed by re-computing the respective analysis with randomly selected months replacing the actual fire months. For each grid cell, we randomly select one month within the same season (i.e., ± 1 month of the month-of-year) in which the largest fire occurred, and in a different year. Then, the averaged temporal evolution of soil moisture anomalies is computed as in Figs. 2 and 3. This is repeated 1,000 times with different random months, respectively. The resulting 5^th^-95^th^ percentile range is then used to assess the statistical significance of observed anomalies shown in Fig. 2 which is diagnosed for observed estimates outside this range.

### Datasets

Soil moisture: ESA CCI satellite soil moisture data^27–29^ is obtained from https://www.esa-soilmoisture-cci.org. We use the combined active and passive ESA-CCI v04.4 product which is obtained by merging information from multiple satellites. The data is available at a 0.25° × 0.25° resolution, with daily temporal resolution.

Vegetation optical depth (VOD): VOD is used as a proxy of biomass. Moesinger et al.^31^ provide VOD at 0.25° × 0.25° resolution derived from Ku-band, X-band, and C-band data by combining information from several satellite sensors (SSM/I, TMI, AMSR-E, AMSR2, WindSat). The X-band product is used for estimating biomass in Fig. 3 and S2 given its availability over the whole study period. Further, in Fig. S4 we use alternative VOD data from Du et al.^51^ who provide a long-term global record of ascending and descending X-band VOD retrievals at a 0.25° grid cell resolution based on AMSR-E and AMSR2 sensors.

Meteorological data: We employ 2 m air temperature, precipitation and net radiation data (the sum of surface net solar radiation and surface net thermal radiation) obtained from the fifth generation ECMWF atmospheric reanalysis of the global climate (ERA5)^52^. ERA5 provides daily data at a 0.25° × 0.25° spatial resolution. To compute the aridity, net radiation is converted into equivalent evaporation in mm by using the latent heat of vaporisation. Then, aridity is defined as the ratio between net radiation and precipitation averaged over the study period.

Population density: We use population density data from the Gridded Population of the World (GPW) Version 4.11^53^ obtained from https://sedac.ciesin.columbia.edu. GPW provides estimates every five years over 2000–2015, but only the 2010 data is used in this study. The data is provided at a spatial resolution of 0.5°.

Land area: We obtain land area estimates on 0.25° × 0.25° grids from the GPW Version 4.11^54^ to compute the burned area fraction per grid cell for Fig. 1. The data is available from https://sedac.ciesin.columbia.edu.

## Data availability

All relevant datasets are publicly available from the references indicated. All data generated and/or analysed during this study are available from the corresponding author on reasonable request.

## Supplementary information

Supplementary file1 (PDF 1844 kb)
