# Supplementary material for: Observational evidence of wildfire-promoting soil moisture anomalies
Source: Sci Rep. 2020 Jul 3;10:11008. doi: 10.1038/s41598-020-67530-4 (PMC7335103; doi:10.1038/s41598-020-67530-4)
Supplement: Supplementary file 1 — Supplementary file1 (PDF 1844 kb) [file 41598_2020_67530_MOESM1_ESM.pdf]

Supplementary material for

Observational evidence of wildfire-promoting soil moisture anomalies

Sungmin O[1], Xinyuan Hou[1], and Rene Orth[1]

[1]Max Planck Institute for Biogeochemistry, D-07745 Jena, Germany

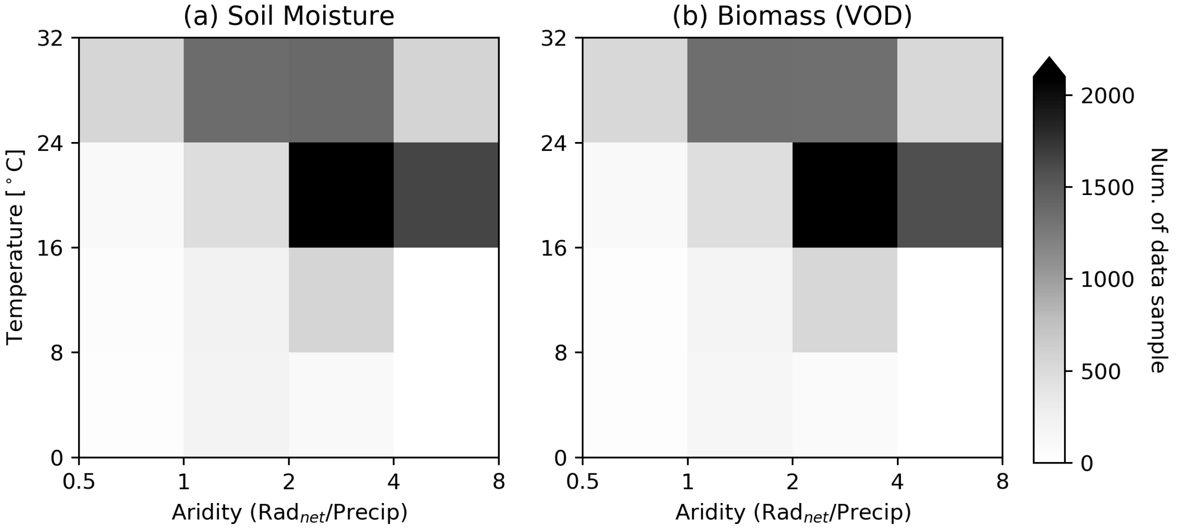

Figure S1: The number of grid cells within each hydro-climatic regime used in Figs. 2 and S2.

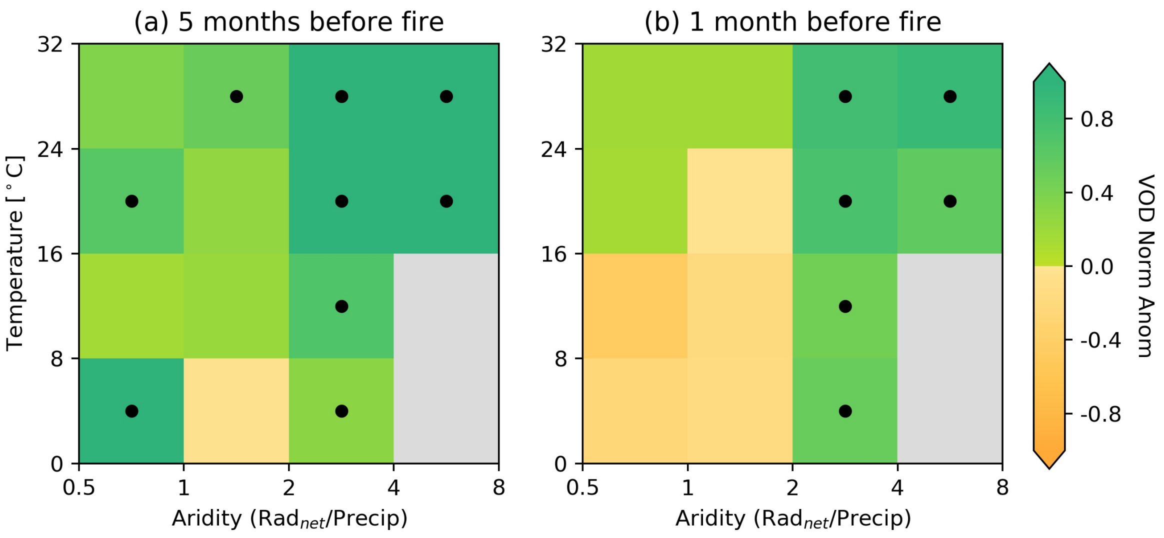

Figure S2: Similar to Fig. 2, but for normalised VOD anomalies. The VOD X-band product from Moesinger et al. (2020) is used.

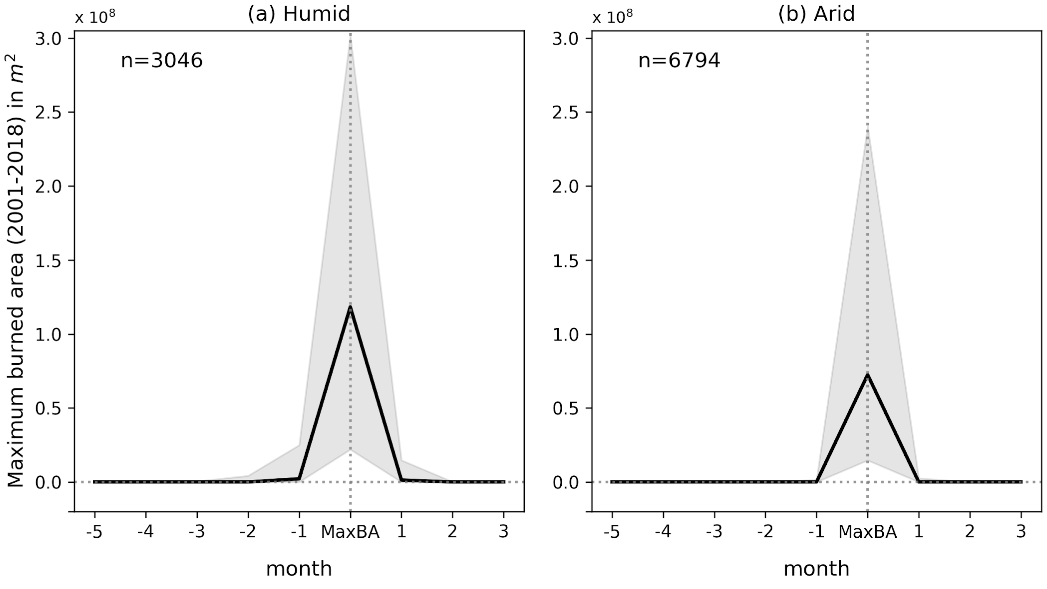

Figure S3: Similar to Fig. 3, but for maximum burned area.

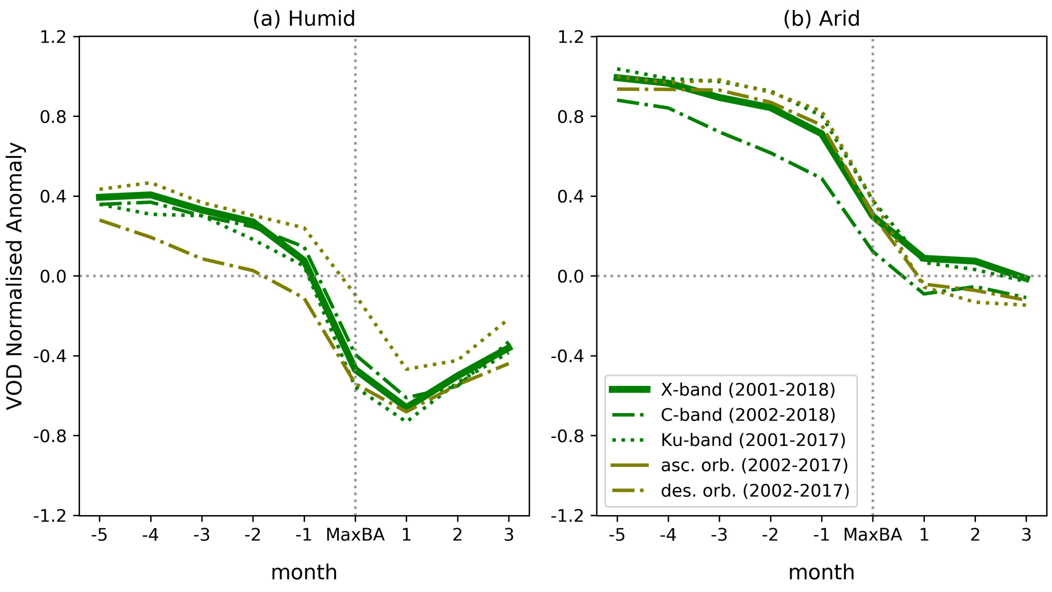

Figure S4: Similar to Fig. 3, but with different VOD products. The thick green line indicates the VOD results shown in Fig. 3, which represent X-band VOD from Moesinger et al. (2020). Ku-Band and C-band VOD from the same product are also shown. In addition, ascending and descending-orbit VOD from multiple sensors processed by Du et al. (2017) are shown. Note that the products are available for slightly different time periods.

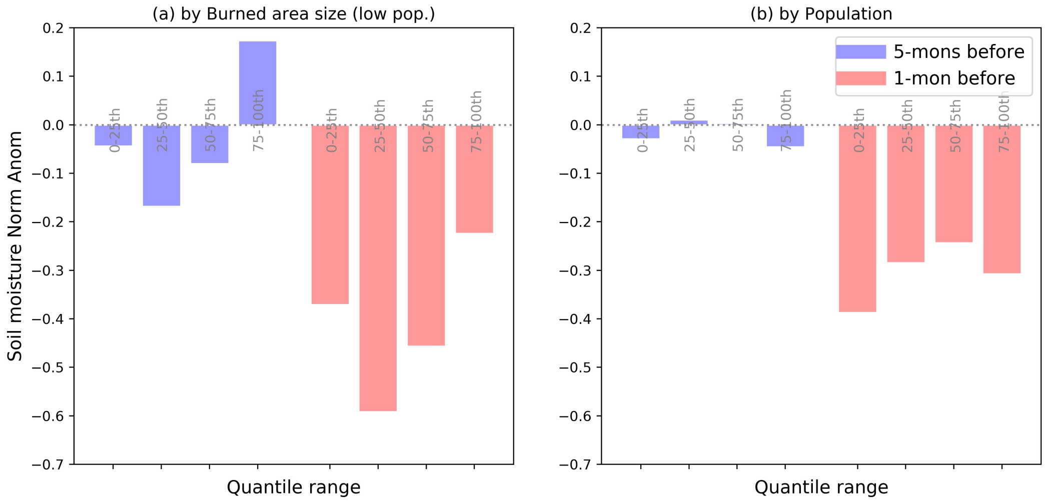

Figure S5: Similar to Fig. 4, but for humid regions with aridity≤2.
